# A two years open-label prospective study of OnabotulinumtoxinA 195 U in medication overuse headache: a real-world experience

**DOI:** 10.1186/s10194-016-0591-3

**Published:** 2016-01-21

**Authors:** Andrea Negro, Martina Curto, Luana Lionetto, Paolo Martelletti

**Affiliations:** Department of Clinical and Molecular Medicine, Sapienza University, Rome, Italy; Regional Referral Headache Centre, Sant’Andrea Hospital, Rome, Italy; Department of Psychiatry, Harvard Medical School, Boston, MA USA; Bipolar & Psychotic Disorders Program, McLean Hospital, Belmont, MA USA; Advanced Molecular Diagnostics Unit, IDI Istituto Dermopatico dell’Immacolata-IRCSS, Rome, Italy; Molecular Medicine Department, Sant’Andrea Hospital, Sapienza University, Via di Grottarossa, 1035-1039 Rome, Italy

**Keywords:** Chronic migraine, Medication overuse headache, Migraine abuse, Preventative therapy, OnabotulinumtoxinA

## Abstract

**Background:**

The efficacy and safety of OnabotulinumtoxinA (BOTOX®) in adults with chronic migraine (CM) were demonstrated in the PREEMPT program. However, the dosage used in this study was flexible from 155 U to 195 U at the physician’s discretion. Therefore, the objective of this prospective study was to compare the efficacy and safety of OnabotulinumtoxinA 195 U vs. 155 U for the treatment of CM and medication overuse headache (MOH) during a 2-year period.

**Methods:**

We prospectively evaluated the mean reduction in headache days, migraine days, acute pain medication intake days and Headache Impact Test (HIT)-6 score in 172 patients injected with OnabotulinumtoxinA 195 U. Successively, we compared the efficacy measures with data of 155 patients injected with OnabotulinumtoxinA 155 U and followed up for 2 years. All patients were affected by CM and MOH, and failed one or more previous detoxification and preventative therapies.

**Results:**

Both OnabotulinumtoxinA 195 U and 155 U reduced significantly the number of headache and migraine days, acute pain medication intake days and HIT-6 score, when compared with the baseline measures. Nevertheless, OnabotulinumtoxinA 195 U proved to be superior of 155 U in all efficacy measures since the first injection and for all the 2 years of treatment, with the exception of the reduction in pain medication intake days that resulted significantly larger with 195 U only after the 4th injection. The safety and tolerability of the two doses were similar and treatment related adverse events were transient and mild-moderate.

**Conclusions:**

This study represents the largest and longest post-marketing studies of doses comparison with OnabotulinumtoxinA in a real-life clinical setting.

Here, we demonstrate the superior efficacy of OnabotulinumtoxinA 195 U compared to 155 U in CM patients with MOH during a 2-year treatment period with similar safety and tolerability profile.

**Electronic supplementary material:**

The online version of this article (doi:10.1186/s10194-016-0591-3) contains supplementary material, which is available to authorized users.

## Background

Migraine has a large individual and socioeconomic burden of disease and, globally, is currently considered the sixth most influential disabling condition according to World Health Organization [1, 2].

Chronic migraine (CM) is a complex and debilitating neurological disorder with a prevalence ranging 1–3 % of the general population and an incidence estimated to be 2.5 % per year [3].

Patients with CM are more likely to have a lower health-related quality of life (HRQoL), greater lost productive time, and greater healthcare resource utilization than episodic migraine patients [4–6].

A significant proportion of patients with CM have a high intake of abortive medications. It is estimated that around 50–80 % of patients with CM referring to headache clinics show analgesic overuse that may lead to the development of medication overuse headache (MOH) [7]. There is not a complete agreement whether MOH is a consequence or a cause of CM [8].

In presence of MOH, prophylactic treatment will be more effective after an adequate withdrawal from the overused medication [9, 10]. At present, prophylactic treatment options are limited and only around the 33 % of CM patients report to be treated and take prophylactic medications [11].

For decades, antiepileptic drugs (e.g., topiramate, divalproex sodium), antihypertensive agents (e.g., beta blockers, calcium channel blockers, angiotensin-converting enzyme [ACE] inhibitors, aldosterone receptor blockers) and tricyclic antidepressants (e.g., amitriptyline, nortriptyline) have been used “off-label” in CM prevention.

Nowadays, OnabotulinumtoxinA is the unique drug specifically indicated for prophylaxis of headache in adult patients with CM [12]. This indication is based on the result of two large-scale, placebo-controlled, multicenter trials (PREEMPT: Phase 3 REsearch Evaluating Migraine Prophylaxis Therapy) that demonstrated the efficacy, safety and tolerability of OnabotulinumtoxinA (155–195 U) as a prophylactic treatment for CM in adults [13–15].

The OnabotulinumtoxinA administration used in the PREEMPT protocol required the intramuscular injection of 155 U of OnabotulinumtoxinA in 31 sites across 7 head and neck muscles using a “fixed-site, fixed-dose” (FSFD) injection paradigm (each injection was 5 U) [16]. Up to 40 U of additional OnabotulinumtoxinA could have been injected at the physician’s discretion using a “follow the pain” (FTP) strategy in 8 additional sites across 3 head and neck muscles [16].

The mechanisms by which OnabotulinumtoxinA decreases the frequency and intensity of pain attacks in CM patients have not been clearly clarified yet. It is known that the activation of the trigeminovascular system is accompanied by release of vasoactive neuropeptides from the presynaptic nerve terminals around leptomeningeal and pericranial vessels [17]. The local release of these neuropeptides induces vasodilation and neurogenic inflammation [17]. Repeated episodes of activation of the trigeminovascular system can sensitize central pain pathways and lead to migraine chronicization [18]. OnabotulinumtoxinA inhibits the release of neurotransmitters (such as serotonin, noradrenaline, dopamine, Gamma-Amynobutirric Acid [GABA], acetylcholine, glutamate, substance P, and Calcitonin Gene-Related Peptide [CGRP]) from peripheral terminals of primary afferents [19], thereby preventing the neurogenic inflammation. Consequently, the peripheral sensitization of nociceptive nerve fibers is inhibited and indirectly reduces the central sensitization [20]. Moreover, it has been recently hypothesized that the antinociceptive effect could not be only dependent from a peripheral mechanism of action of the toxin, but even centrally mediated and axonal transport-dependent [21].

Our experience with OnabotulinumtoxinA for the prevention of chronic daily headaches started in 2001 [22]. In our Headache Clinic the preventive treatment with OnabotulinumtoxinA was offered to all the adults patients that were affected by CM with or without medication overuse (diagnosis made according with the IHS criteria 2004 revised in 2006) (Headache Classification Subcommittee; Headache Classification Committee) [23, 24]. We offered such treatment only to patients with contraindications or lack of efficacy or tolerability to other preventive drugs (beta-blockers, calcium channel blockers, antiepileptics and antidepressants). We did not offer OnabotulinumtoxinA treatment to patients with co-morbid neuromuscular disorders, psychiatric diseases considered incompatible with such kind of treatment, pregnancy and breast-feeding.

Since 2010, we have strictly followed the injection paradigm proposed in the PREEMPT studies [16] using only the FSFD paradigm (155 U in 31 injection sites) and, since 2012, we adopted as standard the combined paradigm FSFD+ FTP (195 U in 39 injection sites) for all the new patients treated with OnabotulinumtoxinA.

With the except of the PREEEMPT studies, large and long-term studies on OnabotulinumtoxinA efficacy and safety have not been published. Moreover, the PREEMPT studies allowed the variable dose from 155 to 195 U, following the pain localization. In this prospective study, we used the 195 U dose and we compared the efficacy and safety profile for the treatment of CM and MOH with a population treated with 155 U over a 2 years period.

## Methods

The protocol was reviewed and approved by the Ethical Committee of Sant’Andrea Hospital, Sapienza University of Rome. Each patient gave a free, informed consent for the participation in the study and the analysis and publication of the protocol data.

We included in the study all the patients affected by CM with MOH, able to fill diaries without any lack of information, referred to our Headache Clinic between January 2012 and January 2013 and followed up for two years.

All patients overused acute pain medications during the one-month baseline period. Medication overuse was defined as simple analgesics intake on ≥15 days, or other medication types or combination of types intake for ≥10 days, with intake ≥2 days/week from the category of overuse.

We included only patients with criteria for MOH who had failed one or more withdrawal attempt, and all the CM patients who had received and failed other preventive therapies due to lack of efficacy or intolerable side effects. Any patient was allowed to take other preventive oral medication during treatment with OnabotulinumtoxinA.

Patients were treated with OnabotulinumtoxinA injections, in multiple sites, combining the “FDFS” and the “FTP” injection paradigm according to the protocol of the PREEMPT study, at the dosage of 195 U for 39 sites. Every session of local injection was repeated every 3 months (±1 week) during a 2-year period.

Headache days, migraine days, and acute pain medication intake were used as efficacy measures. Baseline data were collected from patients headache diary referred to the previous month before starting OnabotulinumtoxinA, and patients were evaluated every three months at the time of each injection. Every six months patients were asked to fill the Headache Impact Test (HIT)-6, used as a measure of efficacy as well, and the results were compared with the baseline score. During the 24 months all adverse events (AEs), related to the drug, were registered and used as a safety measure.

To evaluate if the 195 U was associate with a different efficacy and tolerability than the 155 U, we compared our outcomes with those from a population of patients treated with OnabotulinumtoxinA 155 U and followed up for 2 years with the same injections and clinical evaluation schedule [25].

Continuous variables are reported as mean ± standard deviation, rates and categorical values are reported as subjects-counts and percentage. In the population treated with OnabotulinumtoxinA 195 U, paired t-Test was used to compare the mean headache days, migraine days, medication intake days and HIT-6 score at baseline and at each cycle of injections after Hartley’s ƒ-Max test to assess equal variance of data. A *χ*^2^ test was used to compare categorical variables.

The comparison between OnabotulinumtoxinA 155 U and 195 U was performed using the Student’s t-Test for the continuous variables and the *χ*2 test for the categorical variables. *p* ≤ 0.05 was considered statistically significant.

## Results

### Demographic and baseline headache characteristics

The initial population included was of 172 patients but only 143 patients completed the 2 years follow-up. Table 1 shows the reasons for OnabotulinumtoxinA discontinuation before 24 months.

**Table 1:** Reasons for OnabotulinumtoxinA discontinuation prior to 24 months

Discontinuations due to:	*n* = 29 (16.9 %)
OnabotulinumtoxinA indepent AEs	7 (4.1 %)
Not effective	5 (2.9 %)
Pregnancy	4 (2.3 %)
Drop out	6 (3.5 %)
Personal reasons	7 (4.1 %)

*AEs* adverse events

Demographic and baseline headache characteristics of the 143 patients who completed the protocol are reported in Table 2, together with the comparison with the same demographic data of 132 patients treated with 155 U.

**Table 2:** Baseline demographics and clinical characteristics of the OnabotulinumtoxinA 155 U e 195 U treated groups

	OnabotulinumtoxinA 155 U (*n* = 132)	OnabotulinumtoxinA 195 U (*n* = 143)	t/*χ*2	*P* value
Mean age, years	43.2 ± 13.5 (range 18–76 years)	44.9 ± 12.7 (range 18–78 years)	1.08	0.289
Female, % (n)	81.8 (108)	79.7 (114)	0.08	0.773
Diagnosis of chronic migraine, years	7.6 ± 4.3 (range 0.5–10)	8.4 ± 4.7 (range 1–12 years)	1.47	0.143
Years from the onset of chronic migraine	10.2 ± 4.8 (range 1–40)	9.3 ± 5.1 (range 1–40 years)	1.50	0.133
Monthly headache days	22.3 ± 4.1	22.2 ± 4.9	0.19	0.850
Monthly migraine days	21.4 ± 4.3	21.6 ± 4.8	0.34	0.734
Monthly pain medication intake days	20.8 ± 4.5	21 ± 5.1	0.31	0.757
Mean HIT-6 score	68.9 ± 4.3	67.9 ± 4.2	1.64	0.102
Patients with severe HIT-6, % (n)	93.9 (124)	95.8 (137)	0.18	0.668

Data are presented as mean ± standard deviation; HIT, Headache Impact Test; Scores of 36–49: little or no impact; 50–55: some impact; 56–59: substantial impact; ≥60: severe impact

The majority of patients were female (79.7 %), with mean age 44.9 years (range 18–78). The average time since onset of CM was 9.3 years (range 1–40) (Table 2).

The 95.8 % of patients had a severe (≥60) HIT-6 score. No statistically significant differences in demographic and clinical characteristics at baseline were found between the 155 U and 195 U groups of patients (Table 2).

### Efficacy of 195 U

The comparison of all pre- and post-treatment outcomes is shown in Table 3. The headache days per month decreased significantly during the period of treatment from the first to the eighth session of therapy (pre 22.2 ± 4.9, post 4.1 ± 1.0; *p* < 0.001).

**Table 3:** Pre- and post-treatment outcomes in OnabotulinumtoxinA 195 U treated group

	Headache days	Migraine days	Medication intake days	HIT-6
Baseline (1st injection)	22.2 ± 4.9	21.6 ± 4.8	21.0 ± 5.1	67.9 ± 4.2
3 months (2nd injection)	14.1 ± 3.4*	13.5 ± 3.6*	13.8 ± 3.2*	--
6 months (3rd injection)	10.2 ± 2.8*	9.7 ± 2.7*	9.9 ± 1.9*	61 ± 3.9*
9 months (4th injection)	7.4 ± 2.2*	6.9 ± 1.6*	7.0 ± 1.6*	--
12 months (5th injection)	5.7 ± 1.7*	5.4 ± 1.2*	5.6 ± 1.4*	56.8 ± 3.8*
15 months (6th injection)	5.4 ± 1.2*	4.8 ± 1.0*	5.1 ± 1.3*	--
18 months (7th injection)	4.9 ± 1.3*	4.5 ± 1.0*	4.7 ± 1.3*	54 ± 4.6*
21 months (8th injection)	4.4 ± 1.2*	4.1 ± 1.0*	4.2 ± 1.4*	--
24 months	4.1 ± 1.0*	3.8 ± 1.0*	3.7 ± 1.3*	49 ± 6.7*

Data are presented as mean ± standard deviation

*p ≤ 0.05

There was also a significant reduction of the migraine days per month during the period of treatment from the first to the eighth session of therapy (pre 21.6 ± 4.8, post 3.8 ± 1.0; *p* < 0.001).

Accordingly, also medication intake days decreased significantly during the period of treatment from the first to the eighth session of therapy (pre 21.0 ± 5.1, post 3.7 ± 1.3; *p* < 0.001).

The mean HIT-6 score decreased significantly during the period of treatment from the first to the last injection (pre 67.9 ± 4.2, post 49 ± 6.7; *p* < 0.001), and the proportion of patients with severe (≥60) HIT-6 score decreased as well (pre 95.8 %, post 15.4 %) (Additional file 1: Table S1). After two years of treatment the mean HIT-6 score was ≤55 (some impact, little or no impact) in 68.2 % of patients and ≤49 (little or no impact) in 40.9 % of patients (data not shown). Notably, all the efficacy measures showed a progressive reduction over the 2 years of treatment, that resulted all significant when compared with baseline measures.

### Comparison between 195 U and 155 U

**Fig. 1:**
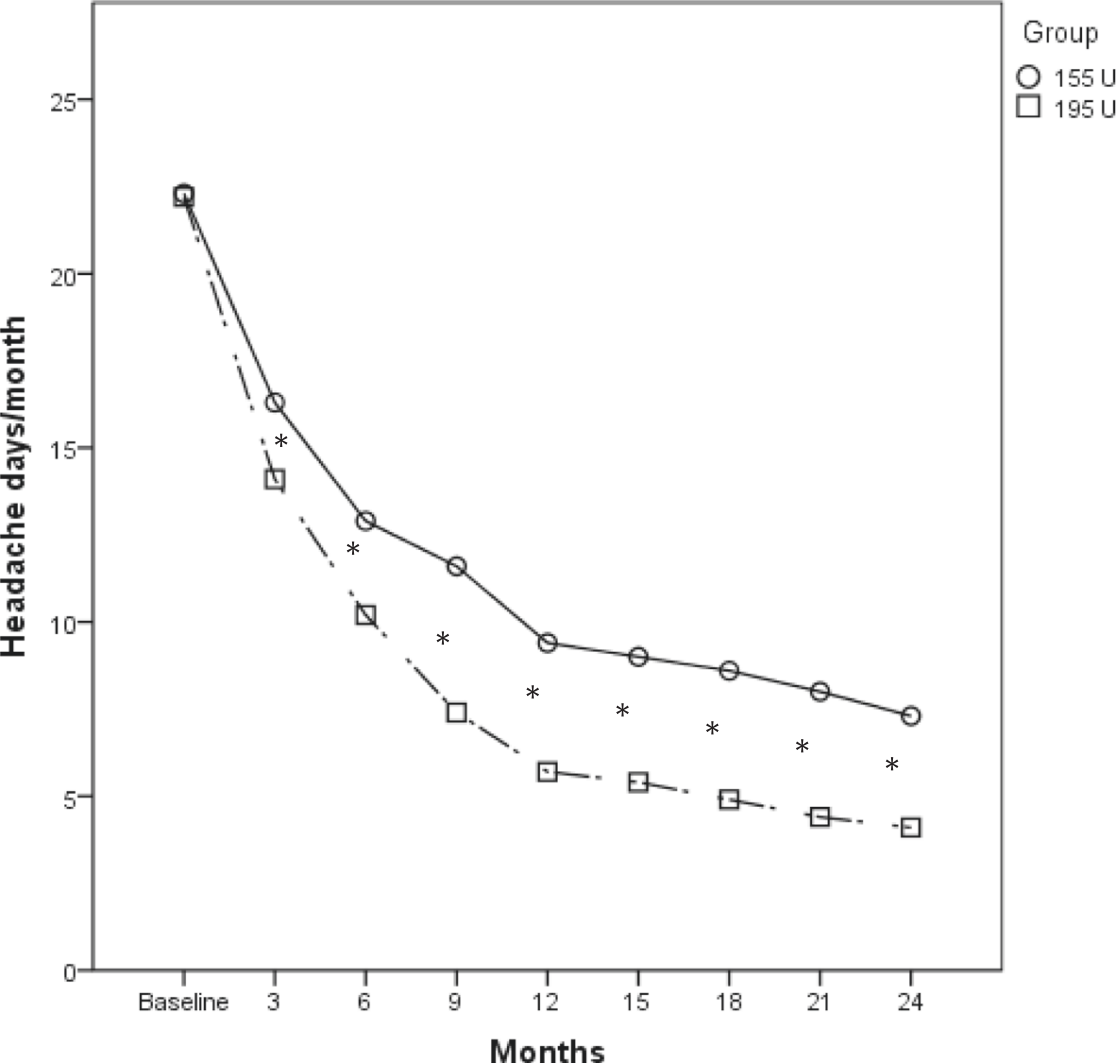
Comparison of the mean change in frequency of headache days in the OnabotulinumtoxinA 155 U and 195 U treated groups. *p < 0.001

**Fig. 2:**
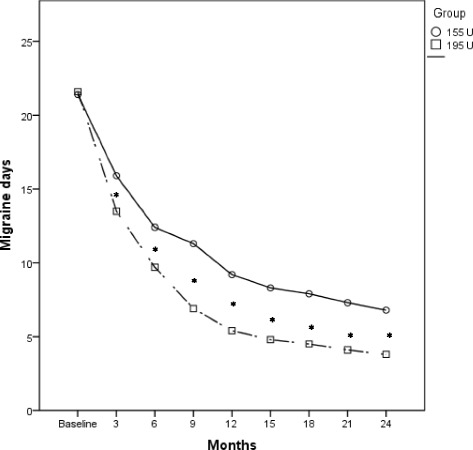
Comparison of the mean change in frequency of migraine days in the OnabotulinumtoxinA 155 U and 195 U treated groups. *p < 0.001

**Fig. 3:**
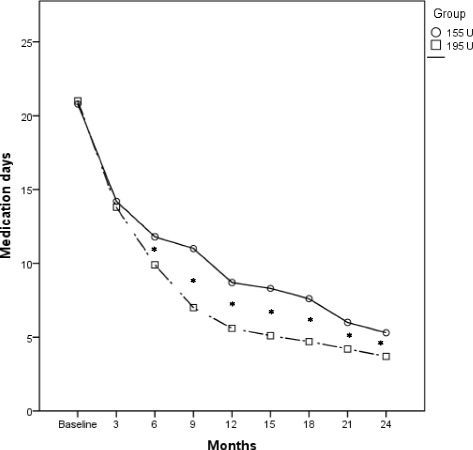
Comparison of the mean change in pain medication intake days in the OnabotulinumtoxinA 155 U and 195 U treated groups. *p < 0.001

**Fig. 4:**
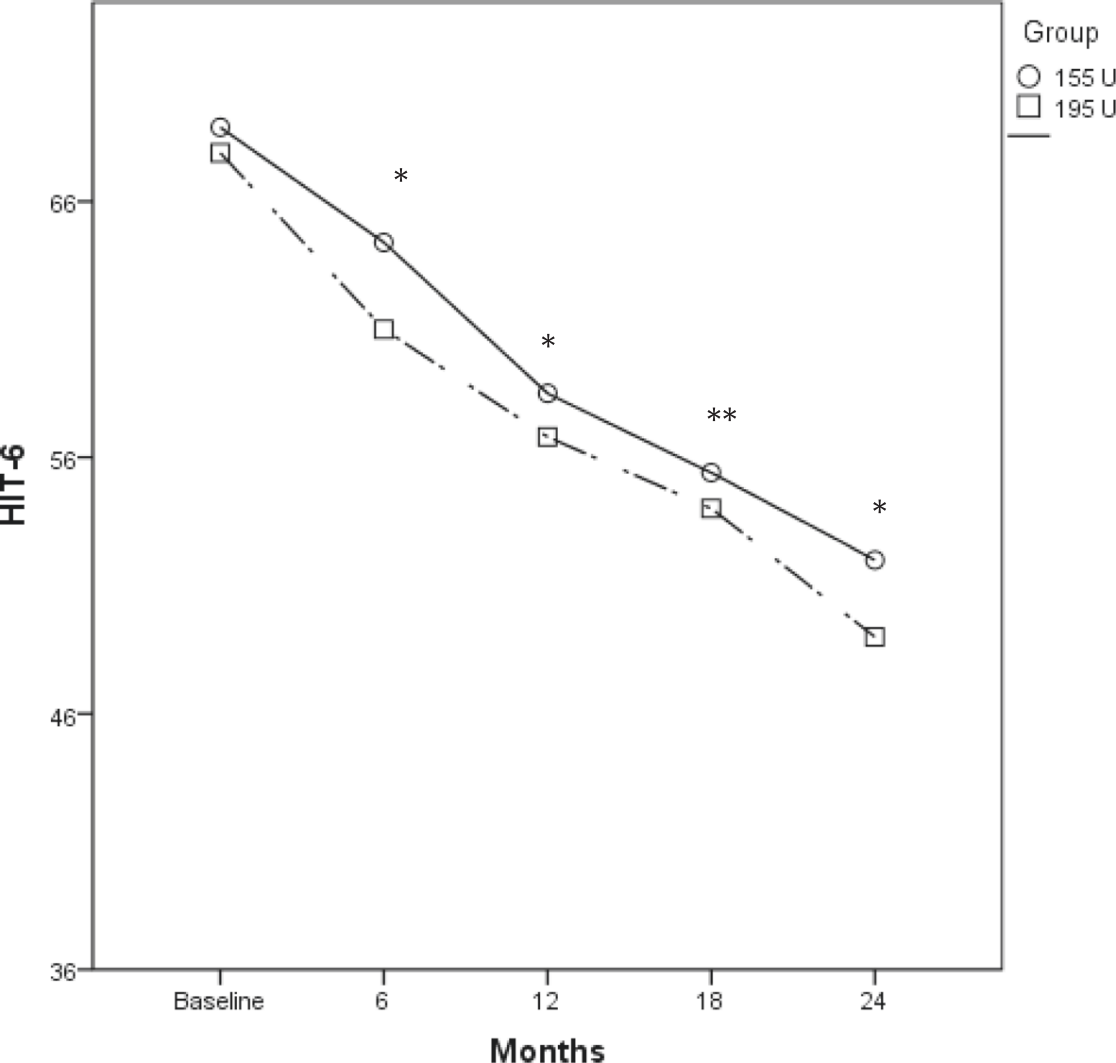
Comparison of the mean change in HIT-6 score in the OnabotulinumtoxinA 155 U and 195 U treated groups. *p ≤ 0.002. **p < 0.05

A graphic comparison of all pre- and post-treatment outcomes in the two populations treated with OnabotulinumtoxinA 195 U and 155 U are shown in Figs. 1, 2, 3 and 4.

Although both dosages were per se effective in reducing headache days frequency since the first injection, OnabotulinumtoxinA 195 U was significantly more effective than the 155 U in reducing the mean headache days at every time point (*p* < 0.001) (Fig. 1, Additional file 1: Table S2).

There was also a significantly larger reduction of migraine days in the group treated with 195 U (*p* < 0.001) (Fig. 2, Additional file 1: Table S3). Moreover, OnabotulinumtoxinA 195 U also significantly decreased medication intake days per month, even if only after the second injection (*p* < 0.001) (Fig. 3, Additional file 1: Table S4).

Similar results were evident also when comparing the mean HIT-6 score between the 195 U and the 155 U treated groups. In fact, OnabotulinumtoxinA 195 U was significantly more effective in reducing the mean HIT-6 score during all the treatment period (*p* < 0.05) (Fig. 4, Additional file 1: Table S5), even if the proportion of patients with severe (≥60) HIT-6 score was not significantly different between the 195 U and the 155 U groups, with the exception of the third injection time (Additional file 1: Table S1).

### Safety and tolerability

Treatment-related AEs were consistent with the known tolerability profile of OnabotulinumtoxinA and were not significantly different between the 195 U and the 155 U treated patients (Table 4). In both groups, the AEs were mild to moderate for severity and persisted for less than a week (e.g., headache, injection-site pain) to a maximum of two months (e.g., eyelid ptosis, cervical musculoskeletal weakness).

**Table 4:** Summary of the overall treatment-related AEs reported in 24 months in the OnabotulinumtoxinA 155 U and 195 U groups

	OnabotulinumtoxinA 155 U (*n* = 132)	OnabotulinumtoxinA 195 U (*n* = 143)	*χ* ^2^	*P* value
	n (%)	n (%)		
Total treatment-related AEs	23 (17.5)	29 (20.3)	0.12	0.728
Injection-site pain	4 (3.3)	5 (3.5)	0.01	0.911
Neck pain	5 (3.8)	6 (4.2)	0.03	0.868
Musculoskeletal weakness	5 (3.8)	7 (4.9)	0.02	0.893
Eyelid ptosis	4 (2.9)	4 (2.8)	0.01	0.911
Headache	5 (3.7)	7 (4.9)	0.02	0.892

*AEs* adverse events

Some AEs occurred more frequently during the first three cycles of injections for both groups (>75 % of all the cases of neck pain and musculoskeletal weakness), whereas others AEs did not show any correlation with the treatment cycle (e.g., headache, eyelid ptosis and injection-site pain) (data not shown).

## Discussion

CM is a serious clinical condition with high risk of medication overuse given by the frequent partial response to treatment, both abortive and preventive. Therefore, the opportunity to provide new effective therapeutic options to patients represents a crucial step in CM treatment [26].

The PREEMPT clinical trials showed that OnabotulinumtoxinA is a safe, well-tolerated, and effective prophylactic therapy for CM patients [13, 14]. To date, the PREEMPT clinical program is the largest (1384 patients) and longest (24-week, double-blind period followed by a 32-week, open-label phase) study in CM [15].

Its remarkable results led to the worldwide specific indication of OnabotulinumtoxinA for the prevention of headache in CM patients [16].

One of the criticisms made to the PREEMPT study design regarded the high percentage (nearly 40 %) of the CM recruited patients that have never received other drugs for migraine prophylaxis. Therefore, in this study we recruited only CM patients that already failed other preventive treatments that were subsequently treated with OnabotulinumtoxinA for 24 months, without taking any other prophylactic treatment.

Several studies have demonstrated that repeated injections over time are able to increase the benefit obtained after the first cycle of treatment, with a prophylactic cumulative effect [27, 28]. In this study, we demonstrated the efficacy and safety of OnabotulinumtoxinA 195 U in the CM with MOH treatment, over a period of 24 months.

Moreover, even if 195 U and the 155 U doses were both effective and safe in the treatment of CM with MOH [25], we found the higher dose being significantly more effective then the 155 U in terms of mean reduction of headache days, migraine days, pain medication intake days and HIT-6 score. Only the severe HIT-6 score proportion of patients did not significantly differ between OnabotulinumtoxinA 195 U and 155 U over the 24 months, with the exception of the second injection time (6 months) in which the 195 U proportion of HIT-6 severe patients resulted significantly lower. These results suggest that the OnabotulinumtoxinA higher dose treatment lead to a faster reduction of the HIT-6 score. The 195 U superior efficacy in all the considered measures was evident since the first injection and was maintained over a period of two years, with the exception of the mean medication intake days reduction that was larger with 195 U only after 6 months.

CM prevention and reduction of attacks might also be essential for the cardiovascular risk reduction, as CM represents an independent risk factor for cardiovascular disorders and ischemic stroke [29]. The reduced attacks frequency implies a greater intake of non-steroidal anti-inflammatory drugs (NSAIDs) and triptans, both associated with a greater likelihood of developing cardiovascular events [29]. Therefore, the greater efficacy of 195 U with respect to 155 U in reducing migraine, headache and medication intake days might be also associated with a larger reduction of cardiovascular risk.

Migraine patients often suffer greatly as a result of the systemic AEs related to the prophylactic drugs, reporting a reduced attention, somnolence, tremor, dizziness, fatigue, depression, loss of appetite, weight gain, hair loss and changes in libido. These side effects are not known in association with OnabotulinumtoxinA.

The safety and tolerability of OnabotulinumtoxinA 195 U were similar to that of OnabotulinumtoxinA 155 U. In our study we enrolled only CM with coexisting MOH patients. We strongly believe that MOH might be a consequence of CM and not just a simple and distinct form of secondary headache [30].

One of the major criticisms made to the PREEMPT studies was about the diagnosis of CM. In fact, in PREEMPT studies, the 65.3 % of patients had medication overuse, which, according to the ICHD-II, precluded the diagnosis of CM [24]. In PREEMPT studies the decision to not to exclude patients overusing acute medications was based on a consultation with the Task Force of the International Headache Society Clinical Trials Subcommittee [31]. In fact, the Task Force guidelines for controlled trials of CM prophylactic treatment in adults recommended the inclusion and stratification of medications overusing patients in clinical studies [32]. Accordingly, a sub-group analysis of the PREEMPT data showed OnabotulinumtoxinA to be equally effective in patients affected by CM with medication overuse [33]. In this study, we provided further evidence in clinical setting of the OnabotulinumtoxinA efficacy for the headache prophylaxis in CM with MOH patients.

Several studies suggest that some migraineurs respond better to OnabotulinumtoxinA than others. A better response has been seen in: CM patients with unilateral headache, pericranial allodynia and pericranial muscle tenderness [34, 35]; those with imploding headache and ocular headache [36]; those with a shorter disease duration (<30 years) [37]; and those with elevated interictal plasma levels of CGRP and vasoactive intestinal peptide (VIP) [38]. Conversely, the prevalence of aura, photophobia, phonophobia, osmophobia, nausea, and throbbing are similar between responders and non-responders [36]. Similarly the presence of white matter lesions at MRI scans did not show the ability to predict a positive response to OnabotulinumtoxinA [39]. In this study, we did not evaluated inter-individual variations in headache features, that might be predictive of a OnabotulinumtoxinA better response, but we can state that the MOH presence do not affect OnabotulinumtoxinA efficacy in CM prophylaxis.

The pharmacologic profile of OnabotulinumtoxinA makes it an appealing candidate for CM patients. Its long duration of action (3 months on average) is attractive for patients with poor compliance to the daily use of preventive medications [40], while its good safety profile makes it the best option when other preventive medications are poor tolerated or non-effective.

OnabotulinumtoxinA mechanism of action is poorly understood. Recent studies have shown that OnabotulinumtoxinA is able to modulate the inflammatory mediators in the trigeminal ganglion [41] and decrease interictal CGRP plasma levels in patients with CM [42], and therefore be effective in the migraine attacks prevention.

There are still questions open in the OnabotulinumtoxinA treatment for CM, such as the possible existence of response predictors (biomarkers, clinical features), the possible OnabotulinumtoxinA paradigm variations depending on the extent of the response, the possible superior efficacy of concomitant oral prophylactic therapies and the optimal management of patients after 2 years of treatment.

## Conclusions

We believe that these results represent the largest and longest post-marketing study of doses comparison with OnabotulinumtoxinA in a real-life clinical setting.

OnabotulinumtoxinA administered at 195 U proved to have indisputable advantages over the 155 U: higher efficacy and same safety and tolerability profile maintained even after a long period of treatment.

On the basis of our results we suggest that the prevention of headache in CM with MOH should be obtained with the higher OnabotulinumtoxinA dosage.

## Additional file

Additional file 1:
**Supplementary Tables.** (DOCX 69 kb)
