# Supplementary material for: A two years open-label prospective study of OnabotulinumtoxinA 195 U in medication overuse headache: a real-world experience
Source: J Headache Pain. 2016 Jan 21;17:1. doi: 10.1186/s10194-016-0591-3 (PMC4720620; doi:10.1186/s10194-016-0591-3)
Supplement: Additional file 1: — Supplementary Tables. (DOCX 69 kb) [file 10194_2016_591_MOESM1_ESM.docx]

| Table S1. Change in percentage of patients with severe impact (HIT-6 score ≥60) in the OnabotulinumtoxinA 155 U and 195 U treated groups. | | | | |
| --- | --- | --- | --- | --- |
|  | **OnabotulinumtoxinA 155 U (n=132)**  **% (n)** | **OnabotulinumtoxinA 195 U (n=143)**  **% (n)** | **χ^2^** | ***P* value** |
| Baseline (1^st^ injection) | 93.9 (124) | 95.8 (137) | 0.18 | 0.668 |
| 6 months (3^rd^ injection) | 77.3 (102) | 63.6 (91) | 5.47 | 0.019* |
| 12 months (5^th^ injection) | 44.7 (59) | 39.9 (57) | 0.48 | 0.491 |
| 18 months (7^th^ injection) | 37.1 (49) | 26.5 (38) | 3.06 | 0.080 |
| 24 months | 22.0 (29) | 15.4 (22) | 1.56 | 0.212 |
| HIT, Headache Impact Test. | | | | |

| Table S2. Comparison of the mean change in frequency of headache days in the OnabotulinumtoxinA 155 U and 195 U treated groups. | | | | |
| --- | --- | --- | --- | --- |
|  | **OnabotulinumtoxinA 155 U (n=132)** | **OnabotulinumtoxinA 195 U (n=143)** | **t** | ***P* value** |
| Baseline (1^st^ injection) | 22.3 ± 4.1 | 22.2 ± 4.9 | 0.19 | 0.850 |
| 3 months (2^nd^ injection) | 16.3 ± 2.7 | 14.1 ± 3.4 | 5.15 | <0.001 |
| 6 months (3^rd^ injection) | 12.9 ± 2.6 | 10.2 ± 2.8 | 6.92 | <0.001 |
| 9 months (4^th^ injection) | 11.6 ± 2.2 | 7.4 ± 2.2 | 13.2 | <0.001 |
| 12 months (5^th^ injection) | 9.4 ± 2.9 | 5.7 ± 1.7 | 11.0 | <0.001 |
| 15 months (6^th^ injection) | 9.0 ± 2.8 | 5.4 ± 1.2 | 12.0 | <0.001 |
| 18 months (7^th^ injection) | 8.6 ± 2.6 | 4.9 ± 1.3 | 12.9 | <0.001 |
| 21 months (8^th^ injection) | 8.0 ± 2.3 | 4.4 ± 1.2 | 13.4 | <0.001 |
| 24 months | 7.3 ± 2.1 | 4.1 ± 1.0 | 14.0 | <0.001 |
| Data are presented as mean ± standard deviation. | | | | |

| Table S3. Comparison of the mean change in frequency of migraine days in the OnabotulinumtoxinA 155 U and 195 U treated groups. | | | | |
| --- | --- | --- | --- | --- |
|  | **OnabotulinumtoxinA 155 U (n=132)** | **OnabotulinumtoxinA 195 U (n=143)** | **t** | ***P* value** |
| Baseline (1^st^ injection) | 21.4 ± 4.3 | 21.6 ± 4.8 | 0.34 | 0.734 |
| 3 months (2^nd^ injection) | 15.9 ± 2.8 | 13.5 ± 3.6 | 5.16 | <0.001 |
| 6 months (3^rd^ injection) | 12.4 ± 2.5 | 9.7 ± 2.7 | 7.34 | <0.001 |
| 9 months (4^th^ injection) | 11.3 ± 2.3 | 6.9 ± 1.6 | 16.0 | <0.001 |
| 12 months (5^th^ injection) | 9.2 ± 2.8 | 5.4 ± 1.2 | 12.2 | <0.001 |
| 15 months (6^th^ injection) | 8.3 ± 3.0 | 4.8 ± 1.0 | 11.1 | <0.001 |
| 18 months (7^th^ injection) | 7.9 ± 3.0 | 4.5 ± 1.0 | 10.8 | <0.001 |
| 21 months (8^th^ injection) | 7.3 ± 2.7 | 4.1 ± 1.0 | 11.1 | <0.001 |
| 24 months | 6.8 ± 2.3 | 3.8 ± 1.0 | 11.9 | <0.001 |
| Data are presented as mean ± standard deviation. | | | | |

| Table S4. Comparison of the mean change in pain medication intake days in the OnabotulinumtoxinA 155 U and 195 U treated groups. | | | | |
| --- | --- | --- | --- | --- |
|  | **OnabotulinumtoxinA 155 U (n=132)** | **OnabotulinumtoxinA 195 U (n=143)** | **t** | ***P* value** |
| Baseline (1^st^ injection) | 20.8 ± 4.5 | 21.0 ± 5.1 | 0.31 | 0.757 |
| 3 months (2^nd^ injection) | 14.2 ± 2.8 | 13.8 ± 3.2 | 0.91 | 0.363 |
| 6 months (3^rd^ injection) | 11.8 ± 2.4 | 9.9 ± 1.9 | 6.29 | <0.001 |
| 9 months (4^th^ injection) | 11 ± 2.1 | 7.0 ± 1.6 | 15.1 | <0.001 |
| 12 months (5^th^ injection) | 8.7 ± 2.7 | 5.6 ± 1.4 | 10.5 | <0.001 |
| 15 months (6^th^ injection) | 8.3 ± 3.0 | 5.1 ± 1.3 | 9.85 | <0.001 |
| 18 months (7^th^ injection) | 7.6 ± 2.9 | 4.7 ± 1.3 | 9.12 | <0.001 |
| 21 months (8^th^ injection) | 6.0 ± 2.3 | 4.2 ± 1.4 | 6.81 | <0.001 |
| 24 months | 5.3 ± 1.7 | 3.7 ± 1.3 | 7.05 | <0.001 |
| Data are presented as mean ± standard deviation | | | | |

| Table S5. Comparison of the mean change in HIT-6 score in the OnabotulinumtoxinA 155 U and 195 U treated groups. | | | | |
| --- | --- | --- | --- | --- |
|  | **OnabotulinumtoxinA 155 U (n=132)** | **OnabotulinumtoxinA 195 U (n=143)** | **t** | ***P* value** |
| Baseline (1^st^ injection) | 68.9 ± 4.3 | 67.9 ± 4.2 | 1.64 | 0.102 |
| 6 months (3^rd^ injection) | 64.4 ± 5.0 | 61.0 ± 3.9 | 5.36 | <0.001 |
| 12 months (5^th^ injection) | 58.5 ± 3.7 | 56.8 ± 3.8 | 3.24 | 0.002 |
| 18 months (7^th^ injection) | 55.4 ± 4.9 | 54.0 ± 4.6 | 2.09 | 0.038 |
| 24 months | 52.0 ± 5.6 | 49.0 ± 6.7 | 3.38 | <0.001 |
| Data are presented as mean ± standard deviation; HIT, Headache Impact Test | | | | |
